# Supplementary material for: Effects of Cultivar Factors on Fermentation Characteristics and Volatile Organic Components of Strawberry Wine
Source: Foods. 2024 Sep 11;13(18):2874. doi: 10.3390/foods13182874 (PMC11432070; doi:10.3390/foods13182874)
Supplement: Supplementary file 1 [file foods-13-02874-s001.zip › foods-3157143-supplementary.pdf]

Table S1. Concentrations of different VOCs in different cultivars of strawberry juice (A) and wines (B)

A

No.	Compound	Juice (μg kg^{-1})				Threshold (μg kg^{-1})
		ZJ	TCL	TZ	BX	
1	3-Methyl-1-butanol	11.06±2.1^{e}	12.47±0.41^{e}	9.72±3.05^{e}	12.62±12.11^{e}	1000
2	2-Heptanol	181.99±157.99^{b}	1093.06±794.76^{a}	1.85±2.51^{b}	1.5±1.14^{b}	65
3	1-Hexanol	100±0^{a}	100±0^{a}	100±0^{a}	100±0^{a}	8000
4	6-Methyl-1-heptanol	12.94±4.66^{c}	8.23±4.68^{cd}	43.35±6.39^{a}	30.51±4.88^{b}	60
5	3-Methyl-1-butanol- acetate	137.01±7.27^{c}	32.61±7.46^{c}	28.13±9.65^{c}	73.45±17.08^{c}	30
6	(Z)-2-Butenoic acid ethyl ester	3.18±1.46^{b}	62.91±103.91^{b}	20.15±13.1^{b}	485.19±379.74^{a}	2500
7	Hexanoic acid ethyl ester	14.32±5.37^{d}	1.05±0.25^{d}	1.09±1.31^{d}	1.27±0.22^{d}	8000
8	2-Hydroxy-propanoic acid ethyl ester	3.52±2.69^{d}	10.58±3.82^{cd}	35.16±5.26^{b}	50.96±14.76^{a}	NF
9	2-Hexenoic acid ethyl ester	1.96±0.32^{a}	4.86±3.84^{a}	5.09±3.18^{a}	2.69±2.4^{a}	670
10	Octanoic acid methyl ester	327.1±284.97^{a}	45.36±9.61^{b}	13.36±0.91^{b}	80.73±15.32^{b}	800
11	Octanoic acid ethyl ester	241.88±18.7^{d}	67.31±60^{d}	308.56±82.12^{d}	349.25±85.88^{d}	580
12	Butyrolactone	11.48±1.84^{e}	11.65±7.65^{e}	17.88±5.72^{de}	18.71±3.3^{de}	20000
13	Decanoic acid ethyl ester	1.86±0.3^{c}	10.65±13.66^{c}	10.69±14.27^{c}	9.86±3.6^{c}	200
14	Butanedioic acid diethyl ester	196.7±66.49^{ab}	144.13±37.44^{b}	174.48±15.67^{ab}	237.9±73.09^{a}	100000
15	3-Hydroxy-hexanoic acid ethyl ester	25.64±7.6^{b}	26.93±1.73^{b}	123.46±113.03^{a}	42.26±70.74^{ab}	NF
16	Ethyl 9-decenoate	360.64±76.59^{b}	317.3±120.93^{b}	530.23±131.22^{b}	2221.14±702.76^{a}	900
17	Carbamic acid phenyl ester	3.24±3.81^{c}	47.8±14.56^{a}	41.01±7.89^{a}	1.15±0.53^{c}	NF
18	Ethyl (Z)-cinnamate	15.38±4.4^{c}	0.82±0.3^{c}	1.13±0.48^{c}	83.55±24.5^{c}	110
19	Hexadecanoic acid ethyl ester	137.47±8.89^{b}	141.74±63.2^{b}	167.92±49.09^{b}	1038.89±380.79^{a}	1500
20	Acetic acid	24.94±8.7^{b}	24.54±8.98^{b}	22.07±11.99^{b}	16.85±6.32^{b}	20000
21	2-Methyl-propanoic acid	12.76±1.76^{b}	10.37±2.61^{b}	5.28±0.49^{b}	18.57±5.4^{b}	2300
22	Butanoic acid	18.74±2.03^{b}	12.39±20.06^{b}	39.95±6.51^{ab}	65.14±58.17^{a}	1000
23	2-Methyl-butanoic acid	23±5.28^{de}	10.99±2.81^{e}	17.91±3.04^{de}	41.66±10.67^{cde}	3000
24	Hexanoic acid	0.98±0.2^{e}	1.69±1.98^{e}	0.82±0.59^{e}	1.2±0.37^{e}	3000
25	Octanoic acid	32.59±9^{c}	125.19±41.17^{c}	135.62±30.24^{c}	891.28±317.54^{a}	500
26	α-Terpineol	1.07±0.6^{d}	4.42±4.42^{d}	6.08±5.12^{d}	15.77±3.91^{d}	250
27	Hexadecamethyl-cyclooctasiloxane	36.17±34.95^{ab}	66.42±36.08^{a}	15.93±12^{b}	38.53±13^{ab}	NF
28	Methoxy-phenyl-oxime	1.28±0.43^{c}	0.96±0.25^{c}	0.97±0.31^{c}	0.86±0.18^{c}	NF
29	Phenylethyl alcohol	127.17±35.59^{d}	124.58±42.85^{d}	125.45±31.75^{d}	310.08±157.13^{cd}	10000

30	2,4-Di-tert-butylphenol	40.68±1.22^{a}	13.31±4.49^{c}	13.27±4.58^{c}	16.56±2.09^{bc}	NF
31	Benzoic acid	12.67±7.31^{d}	21.95±1.34^{d}	36.47±8.34^{d}	196.92±32.33^{a}	1000
32	4-Methyl-2-hexanone	8.36±0.17^{c}	6.25±1.76^{c}	7.86±0.67^{c}	14.69±2.45^{a}	NF
33	trans-2-Pinanol	188.66±15.79^{b}	96.69±17.6^{b}	252.5±57.95^{b}	2253.65±722.83^{a}	40
34	Benzaldehyde	5.85±0.74^{c}	4.34±1.13^{c}	6.67±1.59^{c}	153.93±55.97^{a}	2000
35	4-Methoxy-2,5-dimethyl-3(2H)-furanone	13.78±1.19^{e}	12.35±0.78^{e}	11.93±0.61^{e}	12.9±0.74^{e}	200
36	Acetophenone	43.57±7.89^{a}	19.06±9.2^{bc}	46.35±14.36^{a}	40.31±27.06^{ab}	65
37	Linalool	39.76±16.23^{c}	9.59±3.03^{c}	51.99±5.87^{c}	662.21±254.72^{a}	25.2
38	Phosphonoacetic Acid, 3TMS derivative	73.7±15.98^{b}	48.06±2.87^{b}	55.53±11.8^{b}	378.21±69.69^{a}	NF
39	cis-α-Bisabolene	4.25±1.63^{c}	0.97±0.22^{c}	0.88±0.69^{c}	0.9±0.54^{c}	45
40	Geranyl vinyl ether	42.09±8.53^{c}	55.5±3.76^{c}	42.93±1.24^{c}	95.46±28.75^{c}	16
41	α-Bisabolol	108.53±8.83^{a}	102.77±3.6^{ab}	91.68±3.84^{cd}	95.22±2.79^{bcd}	30

B

No.	Compound	Wine (μg kg^{-1})				Threshold (μg kg^{-1})
		ZJ	TCL	TZ	BX	
1	3-Methyl-1-butanol	8795.32±263.49^{a}	6707.33±432.69^{c}	7570.6±327.03^{b}	5815.56±1147.64^{d}	1000
2	2-Heptanol	11.42±2.48^{b}	16.42±2.45^{b}	18.47±3.14^{b}	14.08±1.13^{b}	65
3	1-Hexanol	26.99±1.67^{b}	15.59±5.35^{b}	26.12±18.9^{b}	29.92±13.41^{b}	8000
4	6-Methyl-1-heptanol	6.7±4.88^{cd}	0.74±0.39^{d}	1.34±0.24^{d}	1.67±1.64^{d}	60
5	3-Methyl-1-butanol- acetate	596.92±165.41^{a}	341.38±57.5^{b}	560.24±104.68^{a}	19.43±8.26^{c}	30
6	(Z)-2-Butenoic acid ethyl ester	0.83±0.44^{b}	0.9±0.3^{b}	0.93±0.57^{b}	6.01±7.89^{b}	2500
7	Hexanoic acid ethyl ester	664.7±136.29^{a}	489.12±51.86^{b}	653.7±27.75^{a}	228.9±97.98^{c}	8000
8	2-Hydroxy-propanoic acid ethyl ester	19.71±1.33^{c}	19.15±1.06^{c}	16.87±1.1^{c}	14.99±4.51^{c}	NF
9	2-Hexenoic acid ethyl ester	0.87±0.65^{a}	0.98±0.5^{a}	1.39±0.37^{a}	2.43±3.13^{a}	670
10	Octanoic acid methyl ester	22.47±4.57^{b}	16.78±3.08^{b}	23.13±5.23^{b}	12.39±8.08^{b}	800
11	Octanoic acid ethyl ester	1891.28±362.5^{a}	1272.49±77.07^{b}	1661.9±51.22^{a}	879.09±366.79^{c}	580
12	Butyrolactone	67.44±11.43^{a}	43.01±4.35^{b}	33.36±2.64^{bc}	29.23±10.43^{cd}	20000
13	Decanoic acid ethyl ester	138.86±38.39^{a}	89.18±9.16^{b}	134.12±15.58^{a}	80.34±29.6^{b}	200
14	Butanedioic acid diethyl ester	41.82±4.86^{c}	23.02±4.25^{c}	58.47±5.75^{c}	28.04±7.91^{c}	100000
15	3-Hydroxy-hexanoic acid ethyl ester	8.14±7.86^{b}	10.87±2.93^{b}	5.9±3.96^{b}	19.6±5.95^{b}	NF

16	Ethyl 9-decenoate	146.08±28.23^{b}	142.92±2.77^{b}	180.4±32.23^{b}	173.67±30.74^{b}	900
17	Carbamic acid phenyl ester	21.86±0.36^{b}	15.58±0.48^{b}	15.53±0.82^{b}	14.51±0.29^{b}	NF
18	Ethyl (Z)-cinnamate	136.63±16.92^{c}	85.87±12.96^{c}	306.48±8.68^{b}	793±268.87^{a}	110
19	Hexadecanoic acid ethyl ester	30.62±3.31^{b}	21.18±6.15^{b}	50±9.83^{b}	70.53±2.87^{b}	1500
20	Acetic acid	250.57±11.09^{b}	239.44±15.5^{b}	274.06±23.47^{b}	801.08±418.79^{a}	20000
21	2-Methyl-propanoic acid	42.34±7.05^{a}	43.87±0.58^{a}	46.76±2.02^{a}	49.91±17.85^{a}	2300
22	Butanoic acid	31.69±3.12^{ab}	28.67±1.34^{ab}	18.1±14.23^{b}	27.78±22.74^{ab}	1000
23	2-Methyl-butanoic acid	46.87±3.99^{bcd}	76.34±3.88^{b}	74.22±3.46^{bc}	226.47±49.53^{a}	3000
24	Hexanoic acid	339.85±22.73^{b}	239.95±14.88^{d}	295.37±13.47^{c}	473.29±56.85^{a}	3000
25	Octanoic acid	553.24±105.84^{ab}	295.2±249.61^{bc}	189.83±301.19^{bc}	337.72±274.64^{bc}	500
26	α-Terpineol	145.79±35.78^{a}	85.32±1.5^{bc}	59.37±28.88^{c}	103.03±36.97^{b}	250
27	Hexadecamethyl-cyclooctasiloxane	48.04±7.19^{ab}	47.87±12.12^{ab}	46.87±5.31^{ab}	47.39±2.78^{ab}	NF
28	Methoxy-phenyl-oxime	2.56±3.28^{bc}	5.05±4.27^{b}	8.89±0.78^{a}	10.91±0.89^{a}	NF
29	Phenylethyl alcohol	1433.82±539.33^{a}	728±70.21^{b}	739.3±55.62^{b}	557.48±124.66^{bc}	10000
30	2,4-Di-tert-butylphenol	14.08±1.49^{c}	11.4±0.94^{c}	15.25±2.48^{bc}	20.02±2.58^{b}	NF
31	Benzoic acid	180.26±9.9^{ab}	161.66±9.71^{b}	165.31±7.12^{b}	136.89±12.72^{c}	1000
32	4-Methyl-2-hexanone	7±2.76^{c}	9.46±1.63^{bc}	12.65±1^{ab}	9.63±4.62^{bc}	NF
33	trans-2-Pinanol	17.01±0.77^{b}	21.25±1.64^{b}	23.23±2.52^{b}	24.87±0.34^{b}	40
34	Benzaldehyde	97.86±2.16^{b}	72.19±1.05^{b}	60.75±44.46^{b}	68.78±5.03^{b}	2000
35	4-Methoxy-2,5-dimethyl-3(2H)-furanone	20.84±2.66^{d}	48.05±4.03^{b}	42±2.02^{c}	157.56±5.91^{a}	200
36	Acetophenone	13.48±11.14^{c}	8.01±5.31^{c}	2.53±0.23^{c}	4.34±4.42^{c}	65
37	Linalool	111.24±7.38^{c}	129.7±7.68^{c}	183.19±14.88^{c}	381.09±33.04^{b}	25.2
38	Phosphonoacetic Acid, 3TMS derivative	20.72±10.55^{b}	33.59±25.76^{b}	27.66±15.8^{b}	36.95±6.03^{b}	NF
39	cis-α-Bisabolene	13.21±9.4^{b}	1.67±0.17^{c}	4.38±3.45^{c}	72.36±3.25^{a}	45
40	Geranyl vinyl ether	628.12±104.57^{a}	435.18±41.85^{ab}	357.24±296.8^{b}	26.26±7.86^{c}	16
41	α-Bisabolol	98.35±2.14^{bc}	78.14±3.48^{f}	82.03±8.7^{ef}	88.4±0.92^{de}	30

Mean ± standard deviation (n=3) followed by different letters within each row indicate significant differences (Duncan test, 5%).

BX: Snow White, TCL: Sweet Charlie, TZ: Tongzhougongzhu, ZJ: Akihime. "NF" means not found.
